# Exploring Nursing Students’ Experiences of Empathy and User Experiences in an Immersive Virtual Reality Simulation Game: Cross-Sectional Study

**DOI:** 10.2196/62688

**Published:** 2025-02-13

**Authors:** Jaana-Maija Koivisto, Sanna Kämäräinen, Katri Mattsson, Satu Jumisko-Pyykkö, Riikka Ikonen, Elina Haavisto

**Affiliations:** 1 Department of Public Health Faculty of Medicine University of Helsinki Helsinki Finland; 2 Department of Health Sciences/Nursing Science Faculty of Social Sciences Tampere University Tampere Finland; 3 Department of Health and Well-Being Turku University of Applied Sciences Turku Finland; 4 Department of HAMK Tech Häme University of Applied Sciences Hämeenlinna Finland; 5 Tampere University Hospital Tampere Finland

**Keywords:** education, nursing, learning, empathy, virtual reality, simulation, user experience, cross sectional

## Abstract

**Background:**

Empathy is associated with better clinical outcomes and patient-care experiences, and it has been demonstrated that training can improve nursing students’ empathy. The use of virtual reality (VR) as an experiential learning strategy may increase the empathetic behavior of caregivers. Although much research exists on the use of VR in education, there is still little research on learning empathy in nursing education through immersive VR games that include a head-mounted display and hand controllers. In addition, it is important to study both learning and user experiences in nursing education that utilizes VR technology.

**Objective:**

This study aims to explore nursing students’ experiences of empathy and user experiences in an immersive VR simulation game.

**Methods:**

A cross-sectional design was used. A total of 52 graduating nursing students from 3 universities of applied sciences in Finland participated in the study. The immersive VR simulation game employed in the study was played with a head-mounted display and hand controllers. The instruments used were the Basic Empathy Scale in Adults (BES-A) before the VR simulation gaming session and the Comprehensive State Empathy Scale (CSES) and AttrakDiff 2.0 Scale after the session.

**Results:**

The students’ overall level of empathy experienced in the immersive VR simulation game was favorable (CSES; mean 2.9, SD 0.57). Participants who had a higher level of empathy (BES-A) before playing the immersive VR simulation game also experienced slightly more feelings of empathy after playing (CSES). However, the association between the measures was not statistically significant (r=0.187, *P*=.18). The overall empathy (CSES) experienced in the immersive VR simulation game was positively correlated with its subscales. The use of the VR simulation provided a positive user experience in all 4 factors of the AttrakDiff 2.0 Scale. Overall User Experience and Emotion Sharing correlated negatively (r=−0.248, *P*=.042), as did Attractiveness and Emotion Sharing (r=−0.327, *P*=.018). Hedonic Quality Stimulation correlated negatively with Cognitive Empathy (r=–0.279, *P*=.045).

**Conclusions:**

The results of this study indicate that the use of an immersive VR simulation game in nursing education as a means of increasing empathy seems promising and justified. The immersive VR simulation game offered positive user experiences, which further supported the idea of implementing it in education. However, more research is needed on what kinds of VR environments are the most effective in promoting empathy among nursing students. Furthermore, when using VR technology in learning, one should consider that the VR setting must not be too technical but rather simple, straightforward, and predictable.

## Introduction

### Empathy in Nursing

Empathy is a key factor in quality of care and patient-centered care [1]. Greater empathy is associated with better clinical outcomes and patient-care experiences [2]. Empathy is an innate characteristic of the individual, and it is dynamic, constantly evolving, and built under the influence of various personal and environmental factors [3]. Empathy can be considered to include 3 dimensions: affective, cognitive, and behavioral [4,5]. The affective dimension consists of caring and a sincere and unconditional acceptance of each health care client, whereas the cognitive dimension is related to interpersonal sensitivity, intuition, and the ability to understand the point of view of another person [4,5]. The behavioral dimension is associated with altruism and therapeutic relationships, which develop empathy in practice; it can be understood as an intention to respond compassionately to the needs and concerns of another person [4,5].

Health care professionals with a high capacity for empathy work more effectively in favor of promoting the patient’s condition [1,4,6]. The empathetic attitude of health care professionals toward health care users strengthens collaboration between them and increases patient satisfaction and commitment to treatment [1,4]. Furthermore, empathy enables appropriate support for patients, increases patient satisfaction, improves patient outcomes, reduces errors, and leads to better overall care [1,4]. An empathetic professional can understand the needs of health care users because it is easier for patients to bring their problems and thoughts to them [6].

Health care workers have felt that work experience and a strong professional identity strengthen empathy, even during busy and stressful times [3,4,7]. Empathy is also linked to job satisfaction among health care professionals and a reduced risk of burnout, but also to compassion fatigue and depression [4,5]. It has been observed that with a decrease in anxiety and other negative emotions, empathy toward the patient increases [7]. In addition, positive interaction in the work community and with patients and relatives increases empathy momentarily [7].

Characteristics of empathy with high or medium stability, such as a strong capacity for empathy developed in childhood, a work environment that supports empathy, and a strong professional identity, have been found to have protective effects against factors that lower empathy [3]. Factors that negatively affect empathy are increased technology use [8]; feelings of anxiety, fear, or uncertainty; a large number of patients; lack of time; negative interaction; and a lack of training in empathy [4,5,7]. While the importance of empathy for nursing is undeniable, many health care professionals face challenges in adopting an empathic communication model for their actions [5].

A variety of personal factors (such as inherent traits, physiological and mental conditions, and professional identity) and external factors (including the work environment, life experiences, and situational stressors) influence the development of empathy [3]. The value and importance of empathy should be emphasized more in health care education [3,6], and empathy should be developed during nursing education [9]. In the health care sector, a significant decrease in empathy has been observed, especially in education [10]. The decline in empathy has been attributed to many factors, such as changed curricular requirements and tightened time constraints that have led to the prioritization of technical and clinical knowledge over humanistic values such as empathy [11].

Several factors affect nursing students’ empathy, including engaging, efficiency, unpredictable, and burdensome elements. These factors are a combination of personal, patient, and environmental influences [12]. Strategies that may foster empathy in nursing students are enhancing self-esteem, boosting self-efficacy, and improving interpersonal relationships [13]. Research has shown that, without specific training, personal traits and factors related to social and family environments significantly influence the development of empathy in nursing students [14].

Training improves individuals’ empathy [2], and empathy training seems to have an impact on the empathy skills of health care students [4,9,15]. Conversely, a review revealed that educational programs did not increase health care students’ empathic concerns [16]. However, nursing education seems to lack systematic training in empathy, although its importance has been identified [17].

### Virtual Reality in Empathy Education

Experiential learning has shown good results in increasing empathy [17] because it focuses on learning through lived or shared experience, and it has been utilized using simulations, role-playing games, and virtual reality (VR) in teaching [5,10,17,18]. A recent systematic review revealed that the application of different modalities of simulation promotes empathy in nursing education [19], while another systematic review demonstrated that for improving empathy, the most effective interventions were immersive and experiential simulations providing opportunities for reflection [20]. Simulation experiences can reduce the decline in experiencing empathy during training because it gives the student a sense of control and helps identify professional behavior in patient relationships [21]. In VR simulations, students have been more in the role of a patient [5,10,22,23] than a nurse [18]. Research has shown that the most effective simulation exercises for learning empathy are those in which the learner takes on the role of a patient [21]. However, Levett-Jones et al [5] found that campus point-of-view simulations, where students experience the world “through the eyes” of a patient with hemiparesis, positively impacted nursing students’ empathy toward persons with disabilities. Furthermore, they found a greater increase in the empathy levels of participants who played the role of a caregiver in a VR simulation compared with participants in the role of a patient.

The use of VR as an experiential learning strategy may increase the empathetic behavior of caregivers [1]. VR pertains to a computer-generated 3D environment that replicates the facets of the physical world [24]. Within health care education, prevalent VR technologies encompass computer-based simulations, haptic simulators, and head-mounted display (HMD) systems, with HMD being the least frequently used [25]. In HMD systems, the sensations of immersion, presence, and interaction [24] are the most pronounced. The level of immersion is determined by the extent to which the VR system supports the user’s perception and use of their body in VR. High-level immersion commonly refers to a 3D experience with a virtual interface, such as HMD, that provides the user with a wide field of view, high-resolution image, sound, and motion detection [1,26]. The concept of presence in VR refers to the user’s experience of a sense of being in a virtual environment rather than in their real physical environment [26]. The concept of interactivity refers to the interaction between the user and the VR environment [25].

The integration of immersive technologies into education offers novel avenues for nursing education, with their adoption having gained momentum during and after the COVID-19 pandemic. Virtual simulations are useful because they enable the combination of theoretical clinical knowledge and practice through realistic patient situations in online learning [27,28]. Students’ experiences of using VR in learning have been mostly positive, and the teaching method has been considered motivating [10,29]. Havola et al [30] found that students spending more time in a VR simulation with an HMD and hand controllers achieved better learning outcomes in patient scenarios than students spending less time in VR. VR has mainly been perceived as an easy-to-use and functional teaching tool [22]. However, nursing students have experienced some technical difficulties in VR simulations, and therefore prior technical practice is required before entering a VR simulation session [18,31].

In VR simulations, factors affecting empathy include a sense of presence and the illusion of being in the body of another person [15]. A qualitative study describing undergraduate nursing students’ empathy in an immersive VR simulation game found that nursing students experienced empathic concern toward a virtual patient, and they recognized the virtual patient’s emotions and responded to those [18]. A systematic review by Bas-Sarmiento et al [17] found variations in empathy levels regarding the different characteristics of health care students, such as gender, age, or cultural background. They stated that younger participants’ empathy levels were higher compared with older participants, and women’s empathy levels were higher than men’s [17].

In a good user experience (UX), a VR simulation game supports learning while keeping the technology in the background. UX is defined as “a consequence of the user’s internal state […], the characteristics of a designed system […], and the context […] within which interaction occurs” [32]. In effective interactions with technology, users feel that they achieve their goals and that their needs regarding technology in a certain situation are met [33]. UX has 4 main qualities: perceived pragmatic quality (PQ; eg, simple, practical, clearly structured), hedonic quality identification (HQI; eg, stylish, connective, presentable), hedonic quality stimulation (HQS; eg, innovative, inventive, creative), and attractiveness (ATT; eg, pleasant, good, motivating) [33,34]. UX determines the overall judgment of a product, the choices made, and user behavior [35]. In designing a good UX, participatory and human-centered design methods are used, and the evaluation of experience is an essential part of this [36,37]. Recently, Law and Heintz [38] have underlined the need to study both learning and UX in education that utilizes technology.

Although there has been much research on the use of VR in education, there is still little research on learning empathy in nursing education through immersive VR games that include HMDs and hand controllers. The objective of this study was to explore nursing students’ experiences of empathy and UX in a VR simulation. The overall aim was to contribute to the increased discussion related to the educational use of VR by creating new information about learning empathy in nursing education by interacting with virtual patients in a VR simulation. This study is part of a larger research project that aims to develop methods that utilize immersive technology in nursing education and to investigate their impact on students’ competencies. The following research questions were addressed:

- What is the extent to which nursing students experience feelings of empathy in a patient scenario in an immersive VR simulation game?
- What factors are associated with the experience of empathy during an immersive VR simulation game?
- How is nursing students’ UX in an immersive VR simulation game?
- How is UX associated with experiencing empathy during an immersive VR simulation game?

## Methods

### Research Design and Participant Recruitment

A cross-sectional design was used. Purposive sampling [39] was used to recruit undergraduate nursing students in their final academic year from 3 universities of applied sciences (UASs) on the southern, western, and southwestern coast of Finland. Each UAS had a contact teacher who was informed about the study and who helped the research group with organizing the data collection. The researchers did not know the students beforehand and were not involved in their teaching or evaluation. The inclusion criteria were as follows: (1) graduating nursing students, (2) participation in a VR simulation, and (3) voluntary participation in the study. Exclusion criteria were medical conditions (such as migraine) that prevented participation in the VR simulation.

### Immersive Virtual Reality Simulation Game

The immersive VR simulation game used in this study utilized an HMD and VR software featuring audio-visual enhancements, encompassing graphics, animations, and haptic feedback. In addition, game elements, such as points and feedback systems, were used to promote learning in a nursing context [40,41]. Developed using the Unity development platform (Unity Technologies), the game was developed for compatibility with Oculus Quest devices. This configuration enables players to engage with the virtual environment using hand controllers, maneuvering within a simulated hospital setting. The features of the VR simulation game are described in Textbox 1.

**Features of the virtual reality simulation game.**

1. The patient scenario
  - A 59-year-old man with no previous illnesses is transferred from the emergency department to the department of inner medicine.
  - The patient has a deteriorating condition involving pneumonia.
2. Learning goals
  - Assess the patient’s clinical state using the Airway, Breathing, Circulation, Disability, Exposure (ABCDE) approach.
  - Recognize patients care needs.
  - Implement nursing interventions.
3. The technology
  - 3D patient and hospital environments developed with the Unity game engine.
  - Single-player game.
  - Oculus Quest head-mounted display and hand controllers.
4. Interaction
  - The user views with a head-mounted display 360-degree 3D environment.
  - The user moves around the virtual hospital room by natural walking or teleporting. The user interacts with the patient by choosing options with hand controllers from the options menu.
  - The patient responds with multisensory feedback (audio, visual, and physical).

**Figure 1:**
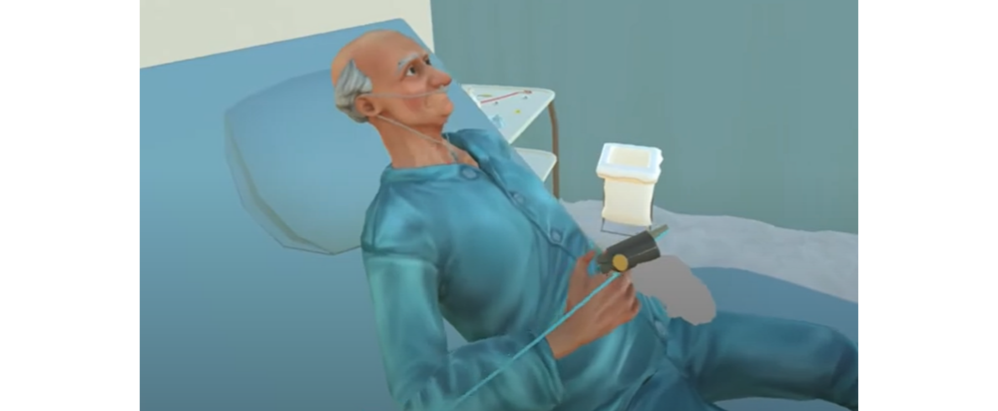
Screenshot of the immersive virtual reality simulation game where a student is placing a pulse oximeter on the virtual patient.

Presented from a first-person view, participants assume the role of a nurse tasked with attending to a virtual patient with pneumonia. Equipped with an Oculus Quest HMD and hand controllers, users conduct clinical assessments and alleviate the patient’s symptoms (Figure 1). Navigation within the virtual hospital room is facilitated through a 360-degree viewing capability, permitting users to either walk naturally or utilize teleportation, a technique employing handheld controllers for movement. The immersive VR simulation game emerged from a collaborative effort across multiple professions within a Finnish UAS. This VR simulation game has been utilized in various studies, including those by Havola et al [30], Mattsson et al [18], and Mäkinen et al [42], demonstrating its efficacy as a learning tool.

### Data Collection

#### Overview

Data were collected by 2 researchers (SK and KM) between May 2021 and February 2022. The individual VR simulation sessions were facilitated by 2 researchers (SK and KM). Students received brief information about the immersive VR simulation game storyline, how to wear the HMD, and how to move and grab things in the virtual world using hand controllers. The researcher verbally instructed students on technical issues if needed when they were immersed in the virtual world. The sessions lasted about 30-45 minutes. The researchers ensured that the gameplay always remained safe by monitoring the situation on-site. They were prepared to end the gameplay if they observed any student experiencing significant dizziness or nausea. However, participants did not report, nor did the researchers observe, any physical adverse effects during the study.

Nursing students filled out an electronic questionnaire before (PRE-Q) and after (POST-Q) the VR simulation session. The PRE-Q included demographic questions (age, gender, educational background, work experience in social and health services, previous gaming activity, and previous experience with VR) and the Basic Empathy Scale in Adults (BES-A) [43]. The POST-Q included the Comprehensive State Empathy Scale (CSES) [5] and the AttrakDiff 2.0 Scale [34]. The instruments used in the study were internationally validated, and permission was obtained for their use in the study. To ensure cross-cultural validation of the instruments, the validation process followed the International Society for Pharmacoeconomics and Outcomes Research (ISPOR) guidelines [44].

#### Basic Empathy Scale in Adults

The BES-A consisted of 20 items [43] assessing nursing students’ empathy with a 5-point Likert Scale from 1 (completely disagree) to 5 (completely agree). Higher scores indicated higher levels of empathy.

#### Comprehensive State Empathy Scale

The CSES [5] measured nursing students’ experience of empathy in the immersive VR simulation game. Students were asked to rate the extent to which they experienced the emotions listed in the CSES in their VR patient scenario. The CSES consists of 6 subscales (Empathic Concern, Distress, Shared Affect, Empathic Imagination, Helping Motivation, and Cognitive Empathy) and 30 items. Each CSES item was scored using a 5-point Likert Scale from 1 (completely disagree) to 5 (completely agree), with higher scores reflecting higher empathy levels.

#### AttrakDiff 2.0 Scale

The AttrakDiff 2.0 Scale measured the UX of the immersive VR simulation game with 4 subscales, including PQ, HQI, HQS, and ATT, and with 28 items evaluated on a 7-point semantic differential scale [34]. AttrakDiff 2.0 is a widely used and validated method for studying UX with interactive products [45-47].

### Statistical Analysis

Data analyses were performed using SPSS Statistics 27.0 (IBM Corp.). In evaluating empathy, descriptive statistics and frequency tables were used to characterize variables. Subscales were formed based on a previously determined instrument structure [5,43]. No severe violations against the prerequisites of parametric tests were observed in skewness and kurtosis evaluations; therefore, parametric tests were chosen: an independent-sample *t* test and 1-way analysis of variance (or Welch test) were used to compare the mean scores of scales and subscales. The correlations between scales and subscales were examined using the Pearson correlation coefficient. The reliability of scales and subscales was evaluated with Cronbach α coefficients.

The AttrakDiff 2.0 questionnaire was analyzed with descriptive statistics. Four factors summarize all their items: (1) PQ (eg, simple/complicated, practical/impractical), (2) HQI (eg, stylish/tacky, isolating/connective), (3) HQS (eg, inventive/conventional, repelling/appealing), and (4) ATT (eg, ugly/attractive, repulsive/inviting) [34]. The internal consistency was acceptable for all factors: PQ (Cronbach α=0.76), HQI (Cronbach α=0.63), HQS (Cronbach α=0.74), and ATT (Cronbach α=0.80). The correlation between UX and CSES evaluations was analyzed using the Pearson correlation coefficient.

### Ethical Considerations

At all stages of the study, the responsible conduct of research guidelines by the Finnish National Board on Research Integrity [48] were followed. Ethical approval was received from the Human Sciences Ethics Committee of Helsinki Region UAS (24.3.2021). In addition, permissions were obtained from the higher-education organizations from which the data were collected. The study participants were informed about the study both orally and in writing. They were told that although playing the game was integrated into their studies, participation in the study was voluntary and that participation did not affect the participants’ grades or academic evaluation. Participants were also informed about the mild physical symptoms such as dizziness, eye fatigue, and nausea that wearing VR headsets may cause. After receiving this information, all participants gave their written consent to participate in the study.

## Results

### User Statistics

A total of 52 graduating nursing students participated in the study (Table 1). Most of the students were aged 21-25 years (27/52, 51.9%). The participants mainly had 1-5 years (22/52, 42.3%) or less than a year (22/52, 42.3%) of work experience in the social and health sector. Most of the participants (43/52, 82.7%) had no previous VR experience in the past year, and no one had used an Oculus Quest headset and hand controllers previously.

**Table 1:** Background variables (n=52).

Characteristics			Values, n (%)
**Gender**			
	Female	42 (81)	
	Male	9 (17)	
	I do not want to tell	1 (2)	
**Age**			
	21-25	27 (52)	
	26-30	15 (29)	
	31-50	10 (19)	
**Educational background**			
	High-school degree	27 (52)	
	Practical nursing	11 (21)	
	Vocational education (other than practical nursing)	3 (6)	
	Bachelor’s degree (other than nursing)	11 (21)	
**Work experience in social and health services**			
	Not at all to 1 year	22 (42)	
	1-5 years	22 (42)	
	Over 5 years	8 (15)	
**Previous virtual reality experience in the past year**			
	Less than once a month	9 (17)	
	Not at all	43 (83)	
**Experience with the Oculus Quest headset and hand controllers**			
	Not at all	52 (100)	

### Nursing Students’ Experiences of Empathy in the Immersive Virtual Reality Simulation Game

The students’ overall level of empathy experienced in the immersive VR simulation game was favorable (CSES; mean 2.9, SD 0.57). The highest level of empathy experienced by nursing students measured by CSES after the VR simulation session was in the subscale Helping Motivation (mean 4.0, SD 0.80), while the lowest were in the subscales Distress (mean 2.1, SD 0.65) and Shared Affect (mean 2.4, SD 0.75; Table 2). At the item level, the highest score was in the item “I found myself thinking about what could be done to help the patient” (mean 4.4, SD 0.85), indicating a high level of empathy. The lowest level of empathy was experienced in the item Upset (mean 1.4, SD 0.64), which, in turn, indicated that the students experienced this emotion in the immersive VR simulation game to a lesser extent.

**Table 2:** Nursing students’ experience of empathy in a virtual reality simulation game (n=52).

Comprehensive State Empathy Scale			Mean (SD)^a^		Cronbach α
**Subscale 1: Empathic Concern**			3.2 (0.74)		0.86
	Compassionate	3.5 (1.16)			
	Moved	2.1 (0.88)			
	Soft-hearted	3.4 (0.93)			
	Sympathetic	3.5 (0.85)			
	Tender	3.6 (0.91)			
	Warm	3.3 (1.07)			
**Subscale 2: Distress**			2.1 (0.65)		0.75
	Distressed	2.5 (1.24)			
	Disturbed	1.8 (0.88)			
	Grieved	1.9 (1.03)			
	Troubled	3.4 (1.05)			
	Upset	1.4 (0.64)			
	Afraid	1.7 (0.85)			
**Subscale 3: Shared Affect**			2.4 (0.75)		0.68
	I found that the scenario affected my mood	3.1 (1.17)			
	I was very affected by the emotions in this story	1.9 (0.93)			
	I actually felt the patient’s distress	2.6 (1.13)			
	I experienced the patient’s feelings as if they were my own	1.9 (0.98)			
**Subscale 4: Empathic Imagination**			2.6 (1.06)		0.87
	I found myself imagining how I would feel in the patient’s situation	2.5 (1.31)			
	I found myself imagining myself in the patient’s shoes	2.2 (1.21)			
	I found myself trying to imagine how things looked to the patient	2.8 (1.28)			
	I found myself trying to imagine what the patient was experiencing	3.0 (1.24)			
**Subscale 5: Helping Motivation**			4.0 (0.80)		0.80
	I would really focus on the patient’s emotions if I were caring for him	3.5 (1.29)			
	I experienced a strong urge to help the patient	3.9 (1.01)			
	I would get really involved in trying to help the patient	4.2 (0.82)			
	I found myself thinking about what could be done to help the patient	4.4 (0.85)			
**Subscale 6: Cognitive Empathy**			2.9 (0.81)		0.86
	I feel confident that I could accurately describe the patient’s experience from his point of view	3.1 (1.01)			
	I found it easy to understand the patient’s reactions	3.4 (0.97)			
	I found it easy to see how the situation looked from the patient’s point of view	3.0 (1.09)			
	Even though the patient’s life experiences are different from mine, I can really see things from their perspective	3.0 (1.07)			
	I am sure that I know how the patient was feeling	2.5 (1.11)			
	I feel confident that I could accurately describe how the patient felt	2.7 (1.08)			
Overall Comprehensive State Empathy Scale score			2.9 (0.57)		0.92

^a^A 5-point Likert Scale with responses ranging from 1 (completely disagree) to 5 (completely agree), with higher scores reflecting higher empathy levels.

### Factors Associated With the Experience of Empathy During the Immersive VR Simulation Game

Participants who had a higher level of empathy (BES-A) before playing the immersive VR simulation game also experienced slightly more feelings of empathy after playing (CSES). However, the association between the measures was not statistically significant (*r*=0.187, *P*=.18). The overall empathy experienced in the immersive VR simulation game was positively correlated with its subscales. The strongest positive correlation in the subscales of empathy was between Empathic Concern and Empathic Imagination (*r*=0.666, *P*<.001; Table 3).

**Table 3:** The correlations between CSES^a^ subscales and BES-A^b^.

Variables	Empathic Concern			Distress			Shared Affect			Empathic Imagination			Helping Motivation			Cognitive Empathy			BES-A		
	*r*	*P* value	*r*		*P* value	*r*		*P* value	*R*		*P* value	*r*		*P* value	*r*		*P* value	*r*		*P* value	
CSES	0.807	<.001	0.466		<.001	0.747		<.001	0.834		<.001	0.623		<.001	0.778		<.001	0.187		.18	
Empathic Concern	—^c^	—	0.164		.24	0.479		<.001	0.666		<.001	0.517		<.001	0.552		<.001	0.270		.05	
Distress	—	—	—		—	0.490		<.001	0.227		.11	0.049		.73	0.145		.31	0.126		.37	
Shared Affect	—	—	—		—	—		—	0.571		<.001	0.367		.007	0.418		.002	0.146		.30	
Empathic Imagination	—	—	—		—	—		—	—		—	0.395		.004	0.631		<.001	0.090		.52	
Helping Motivation	—	—	—		—	—		—	—		—	—		—	0.434		.001	0.240		.09	
Cognitive Empathy	—	—	—		—	—		—	—		—	—		—	—		—	–0.020		.89	

^a^CSES: Comprehensive State Empathy Scale.

^b^BES-A: Basic Empathy Scale in Adults.

^c^Not applicable.

The relationship between the background variables and overall empathy experienced in the immersive VR simulation game is presented in Table 4. Background variables did not have a statistically significant association with the overall empathy experienced in the VR game (Table 4). Examined by subscales, age was associated with cognitive empathy (*F*_2,49_=3.926, *P*=.03) and distress (*F*_2,49_=2.833, *P*=.07) experienced in the game as follows: The experience of cognitive empathy was greater in the 31-50-year age group (mean 3.6, SD 0.78) than in the 21-25- (mean 2.8, SD 0.79) and 26-30-year (mean 2.9, SD 0.73) age groups. Those aged 21-25 years experienced more distress (mean 2.3, SD 0.60) than respondents aged 26-30 (mean 1.9, SD 0.68) or 31-50 (mean 1.9, SD 0.60) years.

When examining the subscales of empathy, the educational background had a weak association with distress experienced in the immersive VR simulation game (*F*_3,48_=2.593, *P*=.06). Students with higher-degree education experienced more feelings of distress (mean 2.3, SD 0.59) than other participants. Work experience seemed to be related to shared affect during the game (*F*_2,25_=3.307, *P*=.05). Those who had worked for more than 5 years experienced less (mean 2.0, SD 0.43) shared affect in the game than other participants (Table 5).

**Table 4:** The relationship between the background variables and the experience of empathy in the immersive virtual reality simulation game.

Variables			n		Mean (SD)		*t* test/*F* test^a,b^ (*df*)		*P* value
**Gender**							0.137 (49)^c^		.89^c^
	Male	9		2.9 (0.61)					
	Female	42		2.9 (0.57)					
**Age (years)**									.59^a^
	21-25	27		2.8 (0.62)					
	26-30	15		2.8 (0.51)					
	31-50	10		3.0 (0.53)					
**Educational background**							0.441 (3, 48)		.73^a^
	High-school degree	27		2.8 (0.61)					
	Practical nursing	11		2.9 (0.40)					
	Vocational education (other than practical nursing)	3		2.5 (0.35)					
	Bachelor’s degree (other than practical nursing)	11		2.9 (0.67)					
**Work experience in social and health services**							1.274 (2, 18)		.30^b^
	Not at all to 1 year	22		2.7 (0.72)					
	1-5 years	22		3.0 (0.35)					
	Over 5 years	8		2.8 (0.56)					

^a^One-way analysis of variance.

^b^Welch *t* test.

^c^*t* test.

**Table 5:** The relationship between background variables and empathy subscales in a virtual reality simulation game (n=52).

Variables			Empathic Concern							Distress							Shared Affect							Empathic Imagination							Helping Motivation							Cognitive Empathy				
			Mean (SD)		*P* value		*F*/*t* test (*df*)		Mean (SD)			*P* value		*F*/*t* test (*df*)		Mean (SD)			*P* value		*F*/*t* test (*df*)		Mean (SD)			*P* value		*F*/*t* test (*df*)		Mean (SD)			*P* value		*F*/*t* test (*df*)		Mean (SD)			*P* value		*F*/*t* test (*df*)
**Gender**					.91^a^		0.110 (49)					.71^a^		0.373 (49)					.84^a^		0.207 (9)					.86^a^		0.176 (49)					.93^a^		0.093 (49)					.78^a^		0.278 (49)
	Male	3.2 (0.55)						2.0 (0.75)							2.4 (1.14)							2.7 (1.25)							4.0 (0.72)							3.0 (1.00)						
	Female	3.2 (0.79)						2.1 (0.61)							2.4 (0.67)							2.6 (1.04)							4.0 (0.83)							2.9 (0.78)						
**Age (years)**					.55^b^		0.612 (2, 49)					.07^b^		2.833 (2, 49)					.98^b^		0.017 (2, 49)					.41^b^		0.896 (2, 49)					.71^b^		0.345 (2, 49)					.03^b^		3.926 (2, 49)
	21-25	3.1 (0.77)						2.3 (0.60)							2.4 (0.72)							2.5 (1.04)							4.0 (0.83)							2.8 (0.79)						
	26-30	3.4 (0.74)						1.9 (0.68)							2.4 (0.65)							2.5 (1.00)							4.0 (0.63)							2.9 (0.73)						
	31-50	3.3 (0.69)						1.9 (0.60)							2.4 (1.04)							3.0 (1.23)							4.2 (0.97)							3.6 (0.78)						
**Educational background**					.64^b^		0.567 (3, 48)					.06^b^		2.593 (3, 48)					.69^b^		0.492 (3, 48)					.36^b^		1.090 (3,48)					.77^b^		0.379 (3, 48)					.40^b^		1.014 (3, 48)
	High-school degree	3.1 (0.87)						2.3 (0.59)							2.5 (0.65)							2.6 (1.01)							3.9 (0.84)							2.8 (0.83)						
	Practical nursing	3.3 (0.49)						1.8 (0.68)							2.4 (0.85)							2.8 (0.92)							4.2 (0.89)							3.3 (0.52)						
	Vocational education (other than practical nursing)	3.2 (0.50)						1.7 (0.33)							1.9 (0.63)							1.7 (0.95)							3.8 (0.14)							2.9 (0.38)						
	Bachelor’s degree other than practical nursing)	3.4 (0.68)						2.0 (0.67)							2.3 (0.96)							2.8 (1.30)							4.1 (0.73)							3.0 (1.04)						
**Work experience in social and health services**					.23^c^		1.557 (2, 27)					.13^b^		2.240 (2, 19)					.05^b^		3.307 (2, 25)					.31^b^		1.258 (2, 20)					.14^b^		2.235 (2, 17)					.43^b^		0.886 (2, 18)
	0-1 year	3.0 (0.95)						2.2 (0.52)							2.4 (0.93)							2.4 (1.18)							3.8 (0.85)							2.8 (0.99)						
	1-5 years	3.4 (0.56)						2.2 (0.70)							2.5 (0.62)							2.9 (0.93)							4.3 (0.58)							2.9 (0.56)						
	Over 5 years	3.4 (0.34)						1.6 (0.66)							2.0 (0.43)							2.4 (1.05)							4.0 (1.06)							3.3 (0.85)						

^a^*t* test.

^b^One-way analysis of variance.

^c^Welch.

### User Experience in the Immersive VR Simulation Game

**Figure 2:**
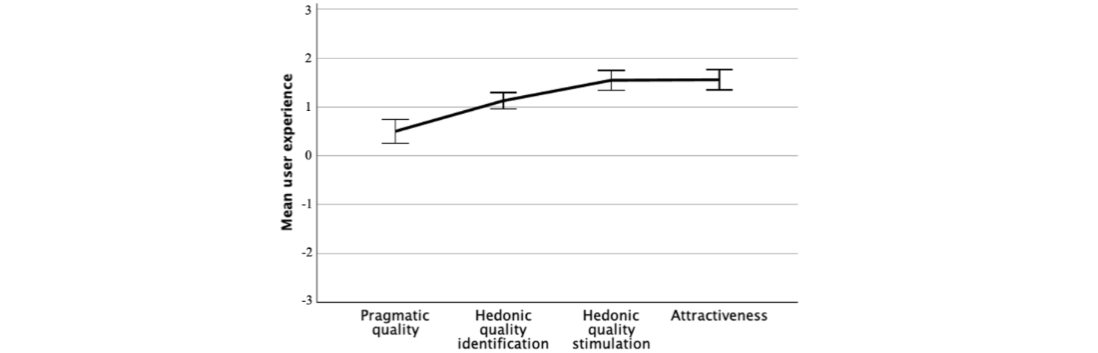
Mean user experience of the virtual reality simulation. The error bars show 95% CI of the mean.

**Figure 3:**
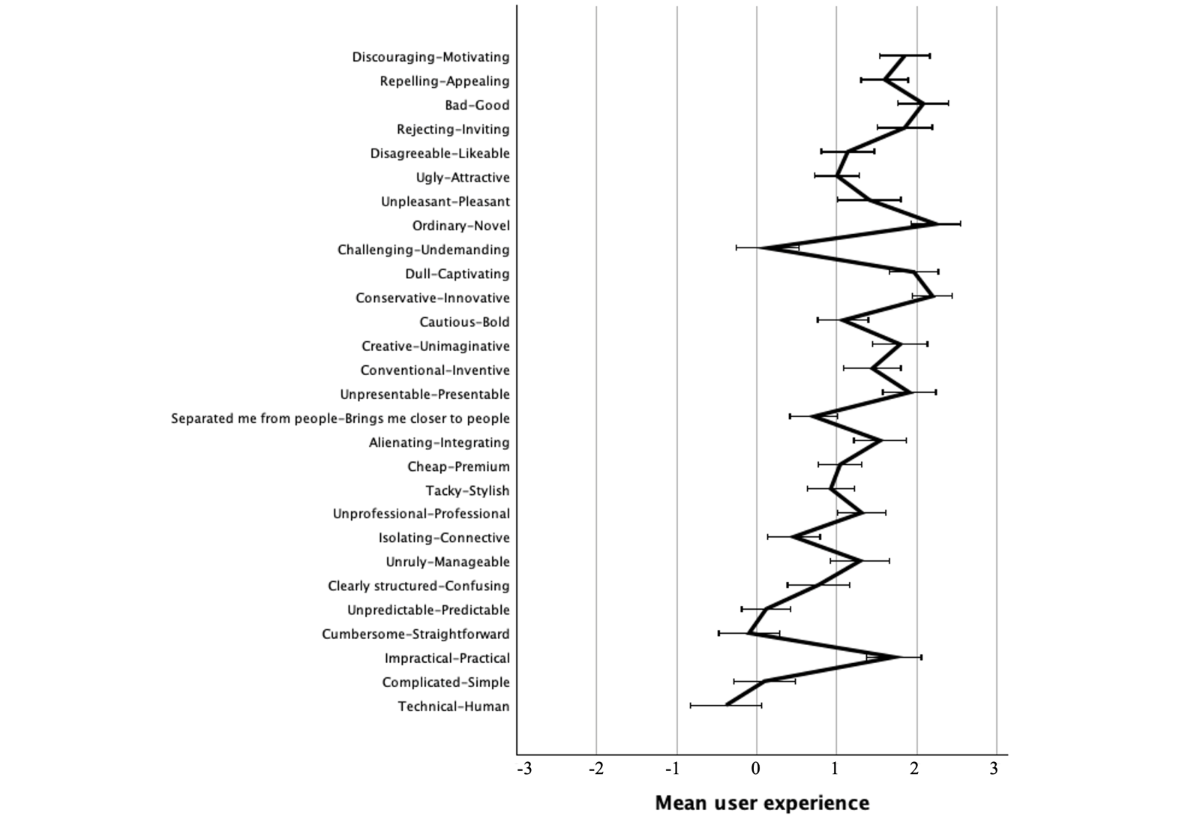
Mean user experience per item of the virtual reality simulation. The error bars show 95% CI of the mean.

The use of the VR simulation provided a positive UX in all 4 factors (Figures 2 and 3). The simulation was experienced as stimulating (mean 1.6, SD 0.72), and this was highlighted in items such as being novel, captivating, or innovative (mean 2.0-2.2, SD 0.87-1.11). However, the VR simulation was experienced as neither challenging nor undemanding (mean 0.1, SD 1.4). The simulation was experienced as being attractive (mean 1.6, SD 0.8) and, in more detail, as being good, motivating, and inviting (mean 1.9-2.1, SD 1.1-1.2). Regarding hedonic quality, the participants were able to positively identify themselves with a situation of simulation (mean 1.1, SD 0.6). This was experienced positively as being presentable, integrating, and professional (mean 1.3-1.9, SD 1.1-1.2). The VR simulation was also mildly positive in its PQ (mean 0.5, SD 0.86). Although the VR simulation was experienced as practical and manageable (mean 1.3-1.9 SD 1.2-1.3), it was seen as slightly technical (mean 0.4, SD 1.6) and neutral in the scales of Complicated-Simple and Cumbersome-Straightforward (mean 0.1, SD 1.4).

### Correlation Between Overall UX and CSES

The correlation between the overall UX and CSES ratings was not statistically significant (*r*=−0.166, *P*=.24, Table 6). In the subscale analysis, overall UX and Shared Affect correlated negatively (*r*=−0.248, *P*=.042), as did ATT and Shared Affect (*r*=−0.327, *P*=.018). HQS correlated negatively with Cognitive Empathy (*r*=–0.279, *P*=.045). All other correlations between the subscales were nonsignificant (Table 6).

**Table 6:** The correlations between CSES^a^ and its subscales and AttrakDiff 2.0 and its subscales^b^.

Variables	UX (AttrakDiff 2.0)			PQ			HQI			HQS			ATT	
	*r*	*P* value	*r*		*P* value	*r*		*P* value	*R*		*P* value	*r*		*P* value
CSES	–0.166	0.240	–0.090		0.524	–0.087		–0.206	0.834		0.143	–0.179		0.205
Empathic Concern	0.039	0.782	0.157		0.267	0.167		0.267	–0.135		0.342	0.011		0.936
Distress	–0.021	0.881	–0.161		0.254	0.098		0.492	0.106		0.454	–0.068		0.631
Shared Affect	–0.284	<0.05	–0.222		0.114	–0.195		0.166	–0.211		0.133	–0.327		<0.05
Empathic Imagination	–0.149	0.293	–0.057		0.689	–0.062		0.661	–0.208		0.139	0.178		0.207
Helping Motivation	–0.105	0.457	0.034		0.813	–0.189		0.181	–0.133		0.349	–0.107		0.448
Cognitive Empathy	–0.166	0.240	–0.166		0.240	–0.159		0.260	–0.279		<0.05	–0.151		–0.179

^a^CSES: Comprehensive State Empathy Scale.

^b^UX measured with AttrakDiff 2.0 User Experience Scale.

## Discussion

### Principal Findings

The purpose of this study was to investigate nursing students’ experiences of empathy by playing an immersive VR simulation game. The overall aim was to contribute to increased discussion related to the educational use of VR by creating new information about learning empathy in nursing education by interacting with virtual patients in a game. The findings presented herein corroborate prior research, indicating a pronounced inclination among nursing students toward the utilization of VR simulation as an educational modality [18].

The main results of this study were that nursing students experienced some feelings of empathy when playing the immersive VR simulation game, which is in line with previous evidence [18,49]. Nursing students experienced the simulation as helping especially motivation and empathic concern. These results are consistent with those of Mattsson et al [18], who showed that nursing students experienced compassion and feelings of concern during a VR simulation game. In addition, the results further support the idea that acting in the role of a nurse might arouse willingness to help the patient and feelings of empathetic concern [5,18].

In this study, playing the immersive VR simulation game clearly evoked more positive than negative emotions in nursing students. Positive emotions experienced by the participants included tenderness, compassion, and sympathy. Negative emotions, such as being upset, afraid, or disturbed, were experienced clearly less, confirming previous study results [18]. This result is positive with regard to learning because feelings of anxiety, insecurity, and fear negatively affect empathy [3,4], whereas a strong professional identity, a sense of control at work, and putting oneself in another person’s shoes increase empathy [3-5,17]. Feeling less distress could be explained by the suitability of the immersive VR simulation game for the stage of the participants’ studies and their current competence level.

The participants felt a strong motivation to help during the immersive VR simulation game. It manifested itself in thinking about what could be done to help the patient, trying to get really involved in helping the patient, experiencing a strong urge to help the patient, and focusing on the patients’ emotions when they were caring for them. These results seem to be consistent with previous research which has indicated that VR simulation increases empathy [1,10,15,29] and represents a usable method for teaching and learning empathy [18]. These results further support the idea that simulation training increases empathy during nursing education and helps identify professional behavior in patient relationships [21].

In our study, nursing students played the role of a nurse in the VR simulation, which differs from many other studies where students have played more the role of a patient [10,22,23]. Beforehand, there has been evidence that the most effective simulation exercises for learning empathy are those in which the learner takes on the role of a patient [21], but the results of this study support the results by Levett-Jones et al [5], who demonstrated an increase in empathy among participants who played the role of a caregiver.

Our results revealed that the participants felt that they understood the patient’s perspective and emotions to some extent, and emotional sharing was fairly limited between the virtual patient and the participants. The cognitive and affective dimensions of empathy can be improved by interventions in which the student represents a specific role, such as the role of a nurse [17], and thus this immersive VR simulation game may not have been optimally successful in promoting the cognitive and affective dimensions of empathy.

Background variables had no statistically significant association with overall empathy (CSES). However, cognitive empathy was greater in the older age group than in the younger age group. In addition, younger participants experienced more distress than older ones. These differences can be explained in part by the life experiences of older participants [3]. Work experience seemed to influence shared affect, such as how the scenario impacted students’ mood and their ability to empathize with the patient’s feelings. Those who had worked for more than 5 years experienced less shared affect than other participants.

The results of the UX assessment showed that the immersive VR simulation game offered a positive UX. Overall, this indicated a good game quality, enabling users to achieve set goals with it, and supporting stimulation, identification, and being attractive. The results also showed that the UX within a simulation can correlate with some aspects of empathy, such as affect sharing and cognitive empathy. The experienced PQ of the VR simulation, and participants’ limited prior experience with VR technology, might have had a small influence on the results. In previous studies conducted with participants with little or no prior experience with VR technology, technology has been reported to capture the participants’ attention to some extent [18,31].

### Limitations

One of the limitations of our study was the frequency of participation in VR simulations. We acknowledge that more frequent participation in VR simulations could potentially yield better and more lasting results. The data set was relatively small for a quantitative study, and therefore, these results need to be interpreted with caution. Usually, cross-sectional studies are quite quick and inexpensive to conduct [50], but our data collection with the immersive VR simulation game during 2021-2022 required the researchers to make considerable practical arrangements due to restrictions set forth by the COVID-19 pandemic and the constant changes it caused in teaching arrangements. This brought about a lot of challenges for data collection. Therefore, the data can be considered reasonable, especially considering that the participants came from 3 different UASs and 3 different cities. However, the relatively small number of participants impacts the reliability of our statistical tests and, consequently, the robustness of our analyses. A larger sample size would provide more statistical power and potentially more reliable and generalizable results. However, as participation was voluntary and the VR simulation sessions were not part of normal teaching, students with an interest in VR were more likely to participate than those not interested in such technology. This could cause sampling bias [50], which could undermine the reliability and generalizability of the study. However, the strength of this study is that multiple outcomes were studied with previously validated instruments, improving the validity of our results. Regarding CSES, there are no previously published research findings on the use of the Finnish version of the instrument. Therefore, this study pilots its application in terms of international equivalence and validity. The values ranged from 0.68 to 0.92, indicating good internal consistency of the instrument.

### Conclusions

The results of this study on the use of an immersive VR simulation game in nursing education as a means of increasing empathy seem promising, as the students mainly experienced positive emotions strengthening their own clinical competence in the VR simulation environment. Based on the results, the use of an immersive VR simulation game to practice empathy skills seems justified in nursing education. However, more research is needed on what kinds of VR environments are the most effective in promoting empathy among nursing students, and on whether playing a VR simulation game repeatedly improves the game’s positive effects on learning. To improve the game in the future, more attention needs to be paid to PQ, such as making the game less technical and more simple, straightforward, and predictable.

## Abbreviations

ATT: attractiveness
BES-A: Basic Empathy Scale in Adults
CSES: Comprehensive State Empathy Scale
HMD: head-mounted display
HQS: hedonic quality stimulation
ISPOR: International Society for Pharmacoeconomics and Outcomes Research
PQ: pragmatic quality
UAS: university of applied sciences
UX: user experience
VR: virtual reality
